# The combination of a seven-autoantibody panel with computed tomography scanning can enhance the diagnostic efficiency of non-small cell lung cancer

**DOI:** 10.3389/fonc.2022.1047019

**Published:** 2022-11-30

**Authors:** Tianyu He, Ziheng Wu, Pinghui Xia, Weidong Wang, Hua Sun, Li Yu, Wang Lv, Jian Hu

**Affiliations:** Department of Thoracic Surgery, the First Affiliated Hospital, Zhejiang University School of Medicine, Hangzhou, China

**Keywords:** NSCLC, nomogram, diagnosis, autoantibodies, EGFR mutation

## Abstract

**Background:**

Non-small cell lung cancer (NSCLC) is still of concern in differentiating it from benign disease. This study aims to validate the diagnostic efficacy of a novel seven-autoantibody (7-AAB) panel for the diagnosis of NSCLC.

**Methods:**

We retrospectively enrolled 2650 patients who underwent both the 7-AAB panel test and CT scanning. We compared the sensitivity, specificity, and PPV of 7-AAB, CT, and PET-CT in the diagnosis of NSCLC in different subgroups. Then, we established a nomogram based on CT image features and the 7-AAB panel to further improve diagnostic efficiency. Moreover, we compared the pathological and molecular results of NSCLC patients in the 7-AABs positive group and the negative group to verify the prognostic value of the 7-AAB panel.

**Results:**

The strategy of a “both-positive rule” combination of 7-AABs and CT had a specificity of 95.4% and a positive predictive value (PPV) of 95.8%, significantly higher than those of CT or PET-CT used alone (P<0.05). The nomogram we established has passed the calibration test (P=0.987>0.05) with an AUC of 0.791. Interestingly, it was found that the 7-AABs positive group was associated with higher proportion of EGFR mutations (P<0.001), lower pathological differentiation degrees (P=0.018), more advanced pathological stages (P=0.040) and higher Ki-67 indexes (P=0.011) in patients with adenocarcinoma.

**Conclusion:**

This study shows that combination of a 7-AAB panel with CT has can significantly enhance the diagnostic efficiency of lung cancer. Moreover, the 7-AAB panel also has potential prognostic value and has reference significance for the formulation of the treatment plan.

## Introduction

According to the latest statistics of cancer incidence and mortality produced by the International Agency for Research on Cancer, lung cancer remained the leading cause of cancer death, with an estimated 1.8 million deaths (18%) worldwide (1). Non–small-cell lung cancer (NSCLC) is the most common pathological pattern of lung cancer, which accounts for over 80% of cases. For NSCLC, the five-year survival rate differs from 92% for patients with stage IA1 disease to only 6% for patients with stage IV disease (2). Therefore, detection of NSCLC at an early stage is critical to improve the overall survival. Regrettably, a large number of patients had been diagnosed with advanced diseases in the past.

Notably, two large randomized controlled trials provided evidence of statistically significant reductions in lung cancer mortality, which benefited from the widespread use of low-dose computerized tomography (LDCT) screening in high-risk populations (3, 4). Although LDCT demonstrates a sensitivity of more than 90% for detecting pulmonary nodules, it still lacks sufficient accuracy to distinguish benign nodules from early-stage lung cancer. So, it leads to high rate of reports of false-positive nodules, as well as unnecessary following-up or surgical procedures. At present, it is urgent to develop a novel examination method to assist CT in improving the diagnostic efficiency of NSCLC.

Due to its non-invasive and reproducible characteristics, serum marker detection can be a good supplement to LDCT. Traditional serum lung cancer biomarkers, such as carcinoembryonic antigen (CEA), squamous cell carcinoma antigen (SCCA) and neuron-specific enolase (NSE), have been clinically used for years. However, they are of limited value in detecting early-stage lung cancer. Tumor-associated antigens (TAAs) exist in most types of cells and are involved in biological processes such as proliferation and differentiation. However, when malignant cells deviate from their normal state, TAAs may be detected by the immune system and generate corresponding autoantibodies (AABs) (5–7). Interestingly, AABs can be detected in an early stage of tumor, especially before significant clinical symptoms appear (8–11). A previous study on a test panel consisting of six serum AABs (p53, NY-ESO-1, CAGE, GBU4-5, Annexin 1 and SOX2) demonstrated a sensitivity/specificity of 36%-39%/89%-91% (12). A prospective study evaluating the EarlyCDT-Lung blood test for differentiating pulmonary nodules between benign and malignant demonstrated that using the “both-positive rule” combination of EarlyCDT-Lung and CT can significantly improve diagnostic specificity (>92%) and positive prediction value (>70%) (13).

In this study, we retrospectively evaluated the diagnostic performance of a 7-AAB panel (p53, PGP9.5, SOX2, GAGE7, GBU4-5, MAGEA1 and CAGE) in lung cancer and compared it with CT and PET-CT for pulmonary nodules with different diameters, stages, imaging features, and pathological types in the same population. At the same time, we summarized the imaging, pathological and molecular characteristics of 7-AAB positive and negative groups in lung cancer patients, suggesting that 7-AAB may have a unique value in the prognostic prediction of lung cancer patients.

## Materials and methods

### Patients

**Figure 1:**
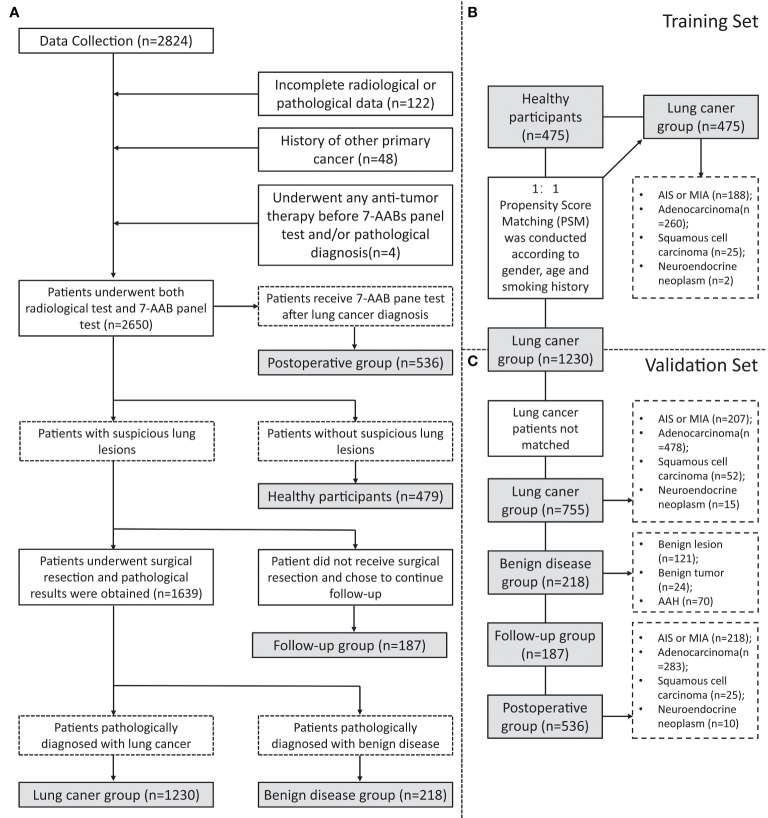
Flowchart of inclusion and grouping. **(A)** A flowchart of the inclusion and exclusion of participants in this study; **(B)** A flowchart of participants in training set matching by Propensity Score Matching (PSM); **(C)** A flowchart of participants inclusion in validation set.

This study included 2824 patients who underwent 7-AAB panel tests in The First Affiliated Hospital, Zhejiang University from January 2020 to April 2022. At the same time, basic clinical information, CT & PET-CT scanning reports (within 1 month before and after the 7-AAB panel test), pathological data, and Next Generation Sequencing (NGS) results were collected. The inclusion and exclusion procedure of patients is shown in **Figure 1**. The CT images are interpreted by at least two experienced radiologists and the final diagnosis is made, as is PET-CT. Patients with pathologically confirmed NSCLC were recommended to undergo NGS detection for their surgically resected tumor specimens according to the clinician’s judgment and the patient’s wishes. NGS testing includes at least the following ten lung cancer-related gene mutations: EGFR (19-DEL, L858R, T790M), KRAS, BRAF, ERBB2, ALK, MET, RET, ROS1, PIK3CA, TP53.

This study was approved by the Medical Ethics Committee and institutional review board of The First Affiliated Hospital, Zhejiang University (Ethical number: IIT20220699A).

### Quantitative detection of serum 7-AABs

The concentrations of serum autoantibodies were quantified by indirect enzyme-linked immunosorbent assay (ELISA) using a detection kit (Cancer Probe Biological Technology Co., Ltd., Hangzhou, China). The procedure of autoantibodies detection was illustrated in **Figure S1**. Briefly, 5ml of venous blood was collected from the patients (using a procoagulant tube), and the upper serum was obtained after centrifugation. The serum samples were diluted with phosphate-buffered saline (PBS) in a ratio of 1:109 and added into antigen-coated microwell plates (50μL/well). The microplate was oscillated and incubated at room temperature for binding of the autoantibodies (primary antibodies) in serum to the pre-incubated TAAs. After washing off the free antibodies with washing buffer, diluted horseradish peroxidase (HRP)-conjugated anti-human IgG (secondary antibody) was added to bind them to the primary antibody. Plates were washed once again using a buffer. Then add chromogenic substrate and incubate with oscillation at room temperature under dark conditions. After the reaction was terminated, the OD450 value was measured with a spectrophotometer immediately.

Calibration wells and quality control wells were set to draw standard curves. The optimal cutoff values of the 7-AABs were defined as mean OD value ± 3 standard deviations (SDs) of the healthy group in the training set. These cutoff values are also used to verify whether the 7-AAB panel is positive or not in the validation set.

### Statistical analysis

Continuous variables were represented by mean ± SD and analyzed by t-test, while categorical variables were expressed as Counts (Percentages) and analyzed by chi-square (χ2) test or Fisher exact test. Sensitivity, specificity, and positive diagnostic value (PPV) were calculated, and the diagnostic value of each diagnostic method was evaluated by area under the curve (AUC) and standard error (SE) for the respective receiver operating characteristic (ROC) curve. A Nomogram was established using logistic regression model and the diagnostic value of this model was evaluated by the calibration curve and ROC curve. For all the analyses, a 2-sided P-value of <0.05 were considered statistically significant. All analyses were performed using SPSS 26.0 software (IBM, Armonk, NY), GraphPad Prism 8.0 Software (GraphPad software, La Jolla, CA), and R 4.1.3 (The R Foundation for Statistical Computing, Vienna, Austria) with the rms statistical package.

## Results

### Study population

To investigate the diagnostic efficiency of the 7-AAB panel, a total of 2650 participants were finally enrolled in this study (**Figure 1A**). They comprised a training set (n=950, **Figure 1B**) and a validation set (n=1698, **Figure 1C**). The baseline clinical characteristics of the patients are summarized in **Tables 1**, **2**. In this study, 475 healthy participants and 475 patients with pathologically-confirmed NSCLC were 1:1 matched (gender, age, and smoking history) using Propensity Score Matching (PSM) in the training set. Meanwhile, 755 patients with NSCLC, 218 patients with benign disease, 187 patients with suspicious lung lesions and continuing follow-up, and 536 postoperative patients (patients receive 7-AAB pane test 6 months to 1 year after surgery) are included in the validation set. Most were females (63.0%), non-smokers (79.9%) and with stage-I disease (80.5%).

**Table 1:** Baseline clinical characteristics of patients in the training set.

Training set			
	Healthy participants (n=475)	Lung cancer (n=475)	P value
Gender, n (%)			
Female	316 (66.5%)	299 (62.9%)	0.248
Male	159 (33.5%)	176 (37.1%)	
Age, years (median, range)	51.2 ± 12.1	51.3 ± 12.3	0.847
Smoking history, n (%)			
Never	392 (82.5%)	374 (78.7%)	0.139
Current/Ever	83 (17.5%)	101 (21.3%)	
Lesion location, n (%)			
Upper left	0	116 (24.4%)	–
Lower left	0	70 (14.7%)	
Upper right	0	157 (33.1%)	
Middle right	0	39 (8.2%)	
Lower right	0	93 (19.6%)	
Pathologic type, n (%)			
AIS or MIA	0	188 (39.6%)	–
IAC	0	260 (54.7%)	
SCC	0	25 (5.3%)	
Neuroendocrine neoplasm	0	2 (0.4%)	
Pathological stage, n (%)			
IA	0	264 (55.6%)	–
IB	0	118 (24.8%)	
IIA	0	19 (4.0%)	
IIB	0	38 (8.0%)	
IIIA	0	8 (1.7%)	
IIIB	0	12 (2.5%)	
IVA	0	10 (2.1%)	
Group of diameters, n (%)			
≤8mm	0	171 (35.7%)	–
8<GGO ≤ 20mm	0	227 (47.4%)	
≥20mm	0	81 (16.9%)	
Number of nodules, n (%)			
Single	0	174 (36.3%)	–
Multiple	0	303 (63.3%)	
Composition, n (%)			
Pure GGO	0	49 (10.2%)	–
Mix GGO	0	305 (63.7%)	
Solid nodule	0	123 (25.7%)	

**Table 2:** Baseline clinical characteristics of patients with surgically resected lung nodules.

Validation set				
	Lung cancer (n=755)	Benign disease (n=218)	Follow-up group (n=187)	Post-operative group (n=536)
Gender, n (%)				
Female	485 (64.2%)	144 (66.1%)	107 (57.2%)	315 (58.8%)
Male	270 (35.8%)	74 (33.9%)	80 (42.8%)	221 (41.2%)
Age, years (median, range)	60 ± 11	54 ± 12	52 ± 15	59.0 ± 11.3
Smoking history, n (%)				
Never	606 (80.3%)	184 (84.4%)	149 (79.7%)	411 (76.7%)
Current/Ever	149 (19.7%)	34 (15.6%)	38 (20.3%)	125 (23.3%)
Lesion location, n (%)				
Upper left	190 (25.2%)	45 (20.6%)	38 (21.6%)	129 (24.1%)
Lower left	125 (16.6%)	39 (17.9%)	15 (8.5%)	85 (15.9%)
Upper right	244 (32.3%)	72 (33%)	64 (36.4%)	160 (29.9%)
Middle right	53 (7%)	18 (8.3%)	13 (7.4%)	48 (9.0%)
Lower right	143 (18.9%)	44 (20.2%)	46 (26.1%)	114 (21.3%)
Pathologic type, n (%)				
Benign lesion	0	121 (56.3%)	0	0
Benign tumor	0	24 (11.2%)	0	0
AAH	0	70 (32.6%)	0	0
AIS or MIA	207 (27.5%)	0	0	179 (33.4%)
IAC	478 (63.6%)	0	0	305 (56.9%)
SCC	52 (6.9%)	0	0	40 (7.5%)
Neuroendocrine neoplasm	15 (2%)	0	0	12 (2.2%)
Pathological stage, n (%)				
IA	586 (77.6%)	0	0	423 (78.9%)
IB	51 (6.8%)	0	0	42 (7.8%)
IIA	31 (4.1%)	0	0	17 (3.2%)
IIB	41 (5.4%)	0	0	23 (4.3%)
IIIA	26 (3.4%)	0	0	20 (3.7%)
IIIB	11 (1.5%)	0	0	8 (1.5%)
IVA	9 (1.2%)	0	0	3 (0.6%)
Group of diameters, n (%)				
≤8mm	182 (24.1%)	98 (45%)	99 (52.9%)	161 (30.0%)
8<GGO ≤ 20mm	364 (48.2%)	88 (40.4%)	72 (38.5%)	256 (47.8%)
≥20mm	209 (27.7%)	32 (14.7%)	16 (8.6%)	119 (22.2%)
Number of nodules, n (%)				
Single	493 (65.3%)	159 (73.3%)	100 (53.5%)	360 (67.2%)
Multiple	262 (34.7%)	58 (26.7%)	86 (46.0%)	176 (32.8%)
Composition, n (%)				
Pure GGO	49 (6.5%)	27 (12.4%)	27 (14.4%)	51 (9.5%)
Mix GGO	419 (55.5%)	86 (39.6%)	114 (61.0%)	318 (59.3%)
Solid nodule	287 (38%)	104 (47.9%)	44 (23.5%)	167 (31.2%)
Spiculation sign, n (%)	459 (60.8%)	88 (40.4%)	61 (32.6%)	295 (55.0%)
Vessels sign, n (%)	560 (74.2%)	97 (44.5%)	74 (39.6%)	374 (69.8%)
Vocule sign, n (%)	302 (40%)	53 (24.4%)	41 (21.9%)	210 (39.2%)
Air bronchogram, n (%)	188 (24.9%)	25 (11.5%)	12 (6.4%)	111 (20.7%)
Pleural indentation, n (%)	231 (30.6%)	30 (13.8%)	16 (8.6%)	142 (26.5%)

### The serum concentration of 7-AABs in the training set

**Figure 2:**
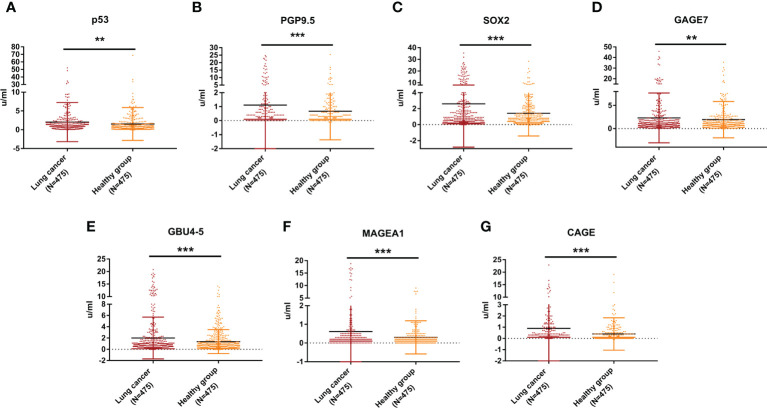
Expression levels of 7-AABs in the training set. **(A–G)** The serum concentration levels of p53, PGP9.5, SOX2,GAGE7, GBU4_5, MAGEA1 and CAGE in lung cancer group and healthy group in the training set, respectively. ** represents p<0.01; *** represents p<0.001.

The serum concentration of the 7 AABs (p53, PGP9.5, SOX2, GAGE7, GBU4-5, MAGEA1 and CAGE) were quantitated by using indirect ELISA. The results showed that the serum concentrations of all 7 AABs in patients with pathologically-confirmed NSCLC were higher than those in healthy participants with statistically significant difference (*P<0.05*) (**Figure 2** & **Table S1**).

### Determination of the cut-off values of the 7-AABs

The optimal cutoff values of the 7-AABs were defined as mean OD value ± 3 standard deviations (SDs) of the healthy participant’s group as previous study described (12). According to the calculation, the cutoff values of p53, PGP9.5, SOX2, GAGE7, GBU4-5, MAGEA1 and CAGE were 17.44 u/ml, 8.16 u/ml, 11.32 u/ml, 15.72 u/ml, 8.52 u/ml, 3.28 u/ml and 5.76, respectively.

### Determination of the diagnostic efficiency of the 7-AAB panel compared with CT scanning or PET-CT in the validation set

The baseline clinical characteristics of patients in the validation set were shown in **Table 2**. The serum concentration of p53, PGP9.5, SOX2, GAGE7, and CAGE were significantly higher in patients with malignant disease than those with benign disease (*P<0.05*, **Table S2**). Different from previous studies, our study observed a significant decrease in serum concentration of p53, PGP9.5, SOX2, GAGE7 and GBU4_5 between the postoperative group compared with the lung cancer group (*P<0.05*, **Table S2**). Therefore, we did not include the postoperative group in the subsequent diagnostic model analysis. In addition, serum concentrations of all 7-AABs except MAGEA1 were found significantly higher in patients with high-risk nodules who were still under radiographic follow-up but had not undergone surgery than in patients with benign disease (*P<0.05*, **Table S2**).

**Figure 3:**
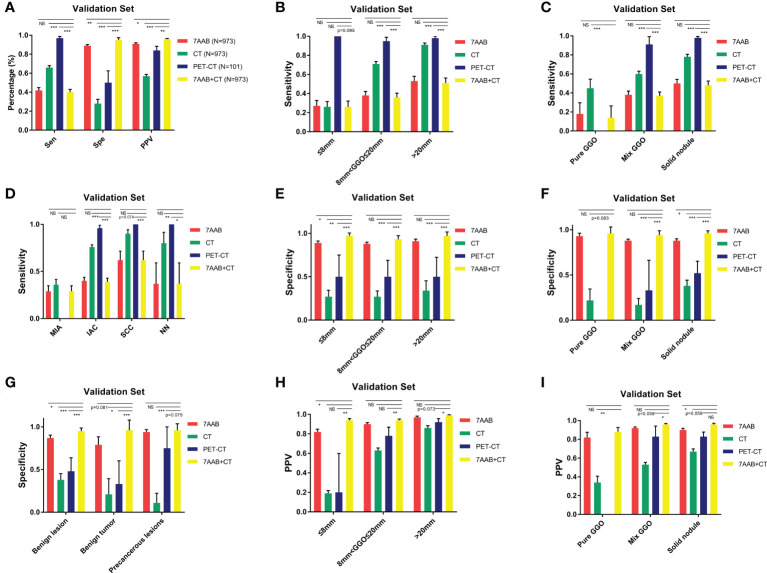
diagnostic efficiency of the 7-AAB panel comparing with CT scanning or PET-CT in different subgroups. **(A)** The sensitivity, specificity and PPV of four different diagnostic methods for diagnosing malignant disease in the validation set; **(B, E, H)** The sensitivity, specificity and PPV of four different diagnostic methods for diagnosing malignant disease in different lesion diameter subgroups, respectively; **(C, F, I)** The sensitivity, specificity and PPV of four different diagnostic methods for diagnosing malignant disease in different lesion composition subgroups, respectively; **(D)** The sensitivity of four different diagnostic methods for diagnosing malignant disease in different pathological type subgroups; **(G)** The specificity of four different diagnostic methods for excluding benign disease in different pathological type subgroups; NS represents no significant difference;* represents p<0.05; ** represents p<0.01; *** represents p<0.001.

We compared the diagnostic efficiency of the single-use 7-AAB panel with CT diagnosis, PET-CT diagnosis, and a “both-positive rule” combination of 7-AABs and CT (shown in **Figure 3**). The result demonstrated the specificity of the 7-AAB panel was higher than both single-used CT diagnosis or PET-CT diagnosis (88.5% vs 28.0% vs 50.0% [*P<0.01*]). The positive predictive value (PPV) of the 7-AAB panel were higher than single-used CT diagnosis and similar to single-used PET-CT diagnosis (90.5% vs 56.8% [*P<0.01*], 90.5% vs 83.3% [*P=0.681*]). Moreover, when combined 7-AAB panel with CT diagnosis, the specificity and PPV reached a higher level (95.4% and 95.8%, respectively). However, the sensitivity of the 7-AAB panel were lower than CT diagnosis and PET-CT diagnosis (41.7% vs 65.7% vs 97.3%, [*P<0.05*]).

In addition, we conducted subgroup analyses to investigate the diagnostic efficiency of the 7-AAB panel in patients with different diameters of lung lesions, composition of radiographic nodule and histological types. In patients with different diameters of lung lesion (≤8mm, 8mm<φ≤20mm, >20mm), a similar trend was observed in all subgroups that the specificity and PPV of the 7-AAB panel were higher than both single-used CT diagnosis or PET-CT diagnosis (shown in **Figure 3** & **Table S3**).

### A nomogram for predicting the probability of malignant disease when combining the 7-AAB panel with patient clinical features in patients with radiological nodules

**Figure 4:**
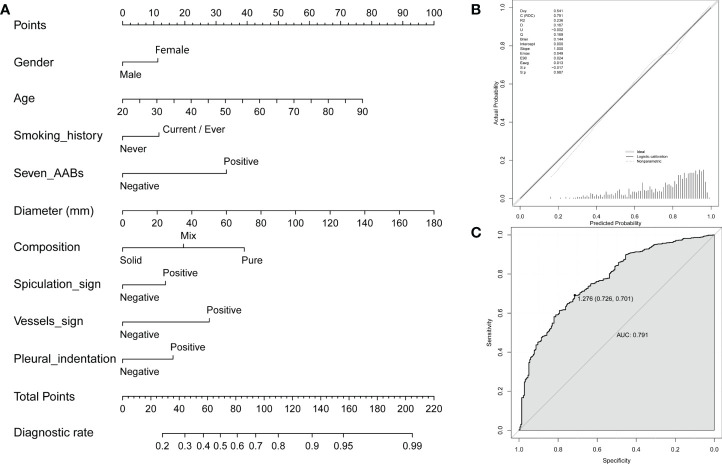
A nomogram for predicting probability of malignant disease. **(A)** A nomogram for predicting probability of malignant disease combining the 7-AAB panel with patient clinical features; **(B)** The calibration curve of the nomogram; **(C)** Receiver operating characteristic (ROC) curve and the corresponding area under the curve (AUC) using the nomogram to predict NSCLC in the training group.

To further optimize diagnostic performance, a nomogram was established based on gender, age, smoking history, 7-AAB panel, and CT imaging characteristics (diameter, composition, spiculation sign, vessels sign and pleural indentation) of patients for predicting malignant disease (**Figure 4A**). Each factor shows a score according to the axis, and the scores of each factor can be added up to obtain an overall score, based on which the probability of malignant disease can be predicted. The corresponding calibration curve of the nomogram is shown in **Figure 4B** and the corresponding ROC curve is shown in **Figure 4C**. The result indicated that this model has passed the calibration test (P=0.987>0.05) and the malignant disease probabilities predicted by the nomogram accorded well with the actual probability, with an AUC of 0.791, showing good discrimination ability. The diagnostic model achieved a sensitivity of 70.1% and specificity of 72.6%, significantly higher than either method used alone.

### The predictive value of the 7-AAB panel for pathological and molecular characteristics of invasive adenocarcinoma and Squamous Cell Carcinoma patients

To investigate the predictive value of the 7-AAB panel in the evaluation of prognosis and subsequent treatment planning, we collected pathological and NGS testing results of pathologically confirmed IAC and SCC patients (shown in **Table 3**). Interestingly, we found that among IAC patients, 7-AAB panel-positive group showed higher proportion of EGFR mutations (81.1% vs 57.6%, P<0.001), compared with lower proportion of ERBB2 mutations (also known as EGFR2, 0.8% vs 6.2%, P=0.020) and ROS1 fusion mutations (0% vs 3.0%, P=0.052). At the same time, the 7-AAB positive group showed lower pathological differentiation degrees (P=0.018), more cases with specific pathological subtype (P=0.067), more advanced pathological stages (P=0.040) and higher Ki-67 indexes (39.15% ± 15.14% vs 26.83% ± 17.58%, P=0.011). To our surprise, a similar trend was not observed in patients with SCC.

**Table 3:** Baseline clinical characteristics of patients with positive or negative 7-AAB test results.

	IAC			SCC		
	7-AABs Positive (n=217)	7-AABs Negative (n=554)	P Value	7-AABs Positive (n=35)	7-AABs Negative (n=42)	P Value
**EGFR mutation, n (%)**	99 (81.1%)	152 (57.6%)	**<0.001**	0	0	–
19 exon Del	39 (39.4%)	69 (45.4%)		0	0	
21 exon L858R	57 (57.6%)	78 (51.3%)		0	0	
20 exon T790M	4 (4.0%)	5 (3.3%)		0	0	
Others	2 (2.0%)	3 (2.0%)		0	0	
**ALK fusion, n (%)**	1 (0.8%)	6 (2.3%)	0.320	0	0	–
**ROS1 fusion, n (%)**	0	8 (3%)	0.052	1 (20%)	1 (7.1%)	0.811
**KRAS mutation, n (%)**	8 (6.6%)	18 (6.8%)	0.925	0	0	–
**BRAF mutation, n (%)**	2 (1.6%)	5 (1.9%)	0.862	0	0	–
**ERBB2 mutation, n (%)**	1 (0.8%)	16 (6.1%)	**0.020**	0	0	–
**MET mutation, n (%)**	1 (0.8%)	6 (2.3%)	0.318	0	0	–
**RET fusion, n (%)**	2 (1.6%)	3 (1.1%)	0.684	0	1 (9.1%)	0.381
**TP53 mutation, n (%)**	17 (13.9%)	40 (15.2%)	0.756	6 (75%)	8 (72.7%)	0.912
**PIK3CA mutation, n (%)**	7 (5.7%)	8 (3%)	0.200	1 (12.5%)	4 (36.4%)	0.243
**Differentiation degree, n (%)**			**0.018**			0.661
High-middle	112 (48.1%)	271 (53.7%)		1 (2.8%)	2 (4.9%)	
Middle	74 (31.8%)	170 (33.7%)		9 (25%)	14 (34.1%)	
Low-middle	26 (11.2%)	45 (8.9%)		16 (44.4%)	13 (31.7%)	
Low	21 (9.0%)	19 (3.8%)		10 (27.8%)	12 (29.3%)	
**Pleural invasion, n (%)**	16 (6.9%)	30 (5.9%)	0.629	2 (5.6%)	4 (9.8%)	0.493
**Vascular cancer embolus, n (%)**	12 (5.2%)	12 (2.4%)	**0.048**	13 (36.1%)	8 (19.5%)	0.103
**Spread through air spaces, n (%)**	6 (2.6%)	16 (3.2%)	0.660	12 (33.3%)	8 (19.5%)	0.168
**Specific pathological subtype, n (%)**	56 (24.0%)	92 (18.2%)	0.067	0	0	–
Micropapillary predominant	24 (42.9%)	41 (44.6%)		0	0	
Solid predominant	25 (44.6%)	42 (45.7%)		0	0	
Cribriform acinar predominant	5 (8.9%)	7 (7.6%)		0	0	
Partial neuroendocrine differentiation	2 (3.6%)	2 (2.2%)		0	0	
**Pathological stage, n (%)**			**0.040**			0.330
IA	167 (77.0%)	439 (79.2%)		5 (13.9%)	11 (26.8%)	
IB	22 (10.1%)	51 (9.2%)		3 (8.3%)	6 (14.6%)	
IIA	8 (3.7%)	23 (4.2%)		1 (2.8%)	3 (7.3%)	
IIB	4 (1.8%)	21 (3.8%)		13 (36.1%)	15 (36.6%)	
IIIA	10 (4.6%)	13 (2.3%)		9 (25%)	3 (7.3%)	
IIIB	2 (0.9%)	5 (0.9%)		4 (11.1%)	3 (7.3%)	
IVA	4 (1.8%)	2 (0.4%)		1 (2.8%)	0	
**Ki-67 positive (%)**	39.15 ± 15.14	26.83 ± 17.58	**0.011**	64.17 ± 26.44	55.00 ± 23.21	0.158

Values in bold represent P values of less than 0.05, indicating statistical significance.

## Discussion

In this retrospective study, we investigated a 7-AAB panel for the diagnosis of NSCLC in a large scale of Chinese population (N=2824), 7-AAB panel test turns out to show superior specificity (88.5%) and PPV (90.5%) to CT scanning and PET-CT scanning for detecting NSCLC. In addition, diagnostic specificity and PPV were further improved when using the “both-positive rule” combination of 7-AABs and CT. This demonstrates the significant value of 7-AABs combined with CT scanning in the diagnosis of NSCLC. And this value has also been verified in lung lesions with different diameters and imaging compositions. Subsequently, we established a nomogram to predict the possibility of malignant disease based on basal information, 7-AAB panel test results, and CT scanning features of patients. Through ROC curve analysis, we proved that it has good predictive performance.

Although the popularization of LDCT screening makes more lung cancer patients diagnosed at an early stage, the low specificity of CT scanning also makes it difficult to distinguish between benign and malignant lesions, especially in patients with pure GGOs and solid pulmonary nodules (14, 15). This may lead to a large number of false-positive reports, which on the one hand occupies more medical resources, and on the other hand, multiple CT examinations also bring potential radiation exposure risks to patients. Therefore, there is an urgent need for a novel diagnostic method to supplement CT scanning in the detection of early lung cancer. Various biomarkers have been used to detect lung cancer, such as circulating tumor cells (CTCs) (16, 17), circulating tumor DNA (ctDNA) (18–20), microbial DNA (mbDNA) (21, 22), microRNA (23, 24), DNA methylation (25) and tumor autoantibodies (12, 26).

In several European studies of EarlyCDT-Lung, the seven-autoantibody panel (p53, NY-ESO-1, GBU4-5, CAGE, SOX2, HuD, and MAGE A4) were confirmed to have 88%-91% specificity and 11%-39% sensitivity (12, 27–29). At present, a number of studies on the use of a 7-AAB panels (p53, PGP9.5, SOX2, GAGE7, GBU4-5, MAGEA1 and CAGE) in the diagnosis of early lung cancer have also been carried out in China. In a prospective study, the sensitivity and specificity of the 7-AAB panel were 61% and 90%, respectively, which were considerably higher than for traditional biomarkers (including CEA, NSE, and CYFRA21-1) (30). In our previous small-scale retrospective study, the 7-AAB panel had a specificity of 90.2% and a PPV of 92.7%, significantly higher than CT scanning. In addition, the utility of the “both-positive rule” combination of 7-AABs and CT has the potential to avoid unnecessary follow-up (31).

In this study, we further increased the sample size and included multiple groups, including the lung cancer group, healthy participants, benign disease follow-up group, and post-operative group. We evaluated the diagnostic performance of the 7-AAB panel compared with CT/PET-CT in multiple dimensions. Compared with our previous study, we got similar results. Since more sample sizes were included in this study and a control group of healthy participants was set, it has a higher reference value. In addition, we redelimited the cutoff value in the training set of a larger sample, because the cutoff value previously used was determined by a researcher in a smaller sample (30).

The 7-AAB panel has similar sensitivity and specificity used alone and has higher specificity and PPV when combining with CT scanning compared to the European Study of EarlyCDT-Lung. Even for solid nodules that were difficult to distinguish, we observed a specificity of 96.0% and a PPV of 96.0% when using the “both-positive rule”. Moreover, a nomogram we established based on basal information, 7-AAB panel test results, and CT scanning features of patients can further improve the accuracy of a lung cancer diagnosis. The diagnostic model achieved a sensitivity of 70.1% and specificity of 72.6%, significantly higher than either method used alone.

Interestingly, by comparing the 7-AABs positive group with the 7-AABs negative group, we found that the 7-AABs positive group was associated with a higher proportion of EGFR mutations and a lower proportion of ROS1 fusion and ERBB2 mutations in IAC patients. This suggests that 7-AABs testing may help screen out potential EGFR mutation patients, that is, patients who may potentially benefit from TKIs treatment and significantly improve their prognosis. One possible explanation is that the activation of EGFR downstream pathway caused by EGFR mutation may lead to the increased expression of some TAAs and the production of detectable autoantibodies. And many studies have confirmed the expression correlation or cascade reaction mechanism between EGFR and SOX2 (32), p53 (33), MAGE-A (34), CAGE (35). The 7-AABs positive group also had lower pathological differentiation degrees, more advanced pathological stages, and higher Ki-67 indexes, which indicate that the tumors in the 7-AABs positive group may have a higher degree of malignancy and a faster proliferation rate. As is known to all, lung cancer patients with EGFR mutation, pathologically low differentiation, advanced pathological stage, and high Ki-67 index have a worse prognosis (36–39), which also suggests the potential prognostic value of the 7-AAB panel test to some extent. A previous study showed that high expression of autoantibodies in lung cancer patients was positively associated with lymph node metastasis and distant metastasis (40). A follow-up study of 264 post-operative patients with NSCLC found that the autoantibody expression level was an independent predictor of poor prognosis (41). Another study of 157 patients with NSCLC showed a five-year survival rate of 62% for the overall population, compared with only 7.6% for those with positive autoantibodies (42).

Unfortunately, due to the short follow-up period, we do not collect enough prognostic data at present. In addition, surprisingly, we did not observe a similar phenomenon in patients with SCC, which may be due to tumor specificity of different pathologic types. Therefore, we will continue to follow up with patients in a long-term study in the future to obtain more prognostic data, to further investigate the relationship between 7AABs and prognosis. In addition, we will include more different pathological types of lung cancer, to better explore the diagnostic and prognostic value of 7-AAB for different types of lung cancer.

## Conclusion

In this study, we verified the diagnostic strategy of a “both-positive rule” combination of 7-AABs and CT scanning in NSCLC that achieved a satisfactory specificity and PPV. In addition, our study developed and validated a novel nomogram based on the 7-AAB panel and CT signature for predicting the risk of NSCLC. Moreover, we revealed that lung adenocarcinoma patients with positive 7-AABs test had a higher ratio of EGFR mutation and worse pathologic features. Taken together, the 7-AABs panel test and our nomogram exhibited robust potential for the diagnosis of NSCLC in clinical practice.

## Data availability statement

The raw data supporting the conclusions of this article will be made available by the authors, without undue reservation.

## Author contributions

Conception and design: TH, ZW, and JH; Data collection: TH, ZW, PX, WW, HS; Analysis and interpretation of data: TH, ZW, PX, LY; Drafting of the manuscript: TH, ZW; Final approval of the manuscript: All authors. All authors contributed to the article and approved the submitted version.

## Funding

This study was funded by Major science and technology special project of Zhejiang Province (2020C03058); Diagnosis and treatment technology research center of pulmonary neoplasm in Zhejiang Province (JBZX-202007) and A Project Supported by Scientific Research Fund of Zhejiang Provincial Education Department (Y202250800).

## Conflict of interest

The authors declare that the research was conducted in the absence of any commercial or financial relationships that could be construed as a potential conflict of interest.

## Publisher’s note

All claims expressed in this article are solely those of the authors and do not necessarily represent those of their affiliated organizations, or those of the publisher, the editors and the reviewers. Any product that may be evaluated in this article, or claim that may be made by its manufacturer, is not guaranteed or endorsed by the publisher.

## Supplementary material

Supplementary Figure 1 Schematic diagram of 7-AABs detection. (A) Procedure for 7-AABs detection by indirect ELISA assay; (B) Setting of patient’s serum samples, loading control samples, calibration samples and quality control samples during 7-AAB panel testing.
