# Supplementary material for: The combination of a seven-autoantibody panel with computed tomography scanning can enhance the diagnostic efficiency of non-small cell lung cancer
Source: Front Oncol. 2022 Nov 30;12:1047019. doi: 10.3389/fonc.2022.1047019 (PMC9748614; doi:10.3389/fonc.2022.1047019)
Supplement: Supplementary file 1 [file Table_1.docx]

**Table S1. The serum concentration of 7-AABs in each group in the training set**

| **Training set** | | | |
| --- | --- | --- | --- |
|  | **Healthy participants (n=479)** | **Lung cancer (n=479)** | **P value** |
| p53, u/ml (median, range) | 1.54±4.36 | 2.04±5.21 | 0.028 |
| PGP9.5, u/ml (median, range) | 0.67±2.04 | 1.11±3.44 | 0 |
| SOX2, u/ml (median, range) | 1.42±2.83 | 2.6±5.4 | 0 |
| GAGE7, u/ml (median, range) | 1.95±3.93 | 2.33±5.39 | 0.029 |
| GBU4_5, u/ml (median, range) | 1.36±2.13 | 1.97±3.64 | 0 |
| MAGEA1, u/ml (median, range) | 0.29±0.82 | 0.62±2.33 | 0 |
| CAGE, u/ml (median, range) | 0.4±1.44 | 0.9±3.45 | 0 |
