# Supplementary material for: The combination of a seven-autoantibody panel with computed tomography scanning can enhance the diagnostic efficiency of non-small cell lung cancer
Source: Front Oncol. 2022 Nov 30;12:1047019. doi: 10.3389/fonc.2022.1047019 (PMC9748614; doi:10.3389/fonc.2022.1047019)
Supplement: Supplementary file 2 [file Table_2.docx]

**Table S2. The serum concentration of 7-AABs in each group in the validation set**

| **Validation set** | | | | | |
| --- | --- | --- | --- | --- | --- |
|  | **Lung cancer (n=888)** | **Benign disease (n=263)** | **Follow-up group (n=187)** | | **Post-operative group (n=536)** |
| p53, u/ml (median, range) | 2.26±5.41 | 1.70±3.40 **^a^** | 2.93±8.15 **^b^** | 1.50±3.30 **^c^** | |
| PGP9.5, u/ml (median, range) | 1.19±3.61 | 0.65±2.03 **^a^** | 0.97±2.79 | 0.82±2.10 **^c^** | |
| SOX2, u/ml (median, range) | 3.01±6.50 | 1.18±1.98 **^a^** | 2.40±5.23 **^b^** | 1.67±4.21 **^c^** | |
| GAGE7, u/ml (median, range) | 3.19±7.54 | 2.06±5.43 **^a^** | 3.54±7.20 **^b^** | 1.94±4.51 **^c^** | |
| GBU4_5, u/ml (median, range) | 2.39±3.94 | 1.78±3.02 **^a^** | 2.80±4.28 **^b^** | 1.77±2.91 **^c^** | |
| MAGEA1, u/ml (median, range) | 0.69±2.90 | 0.46±1.79 | 0.53±2.51 | 0.60±1.90 | |
| CAGE, u/ml (median, range) | 0.75±2.52 | 0.52±1.44 **^a^** | 0.72±2.62 | 0.62±2.54 | |

**^a^** represents a significant statistical difference between this group with Lung cancer group (p＜0.05).

**^b^** represents a significant statistical difference between this group with Benign disease group (p＜0.05).

**^c^** represents a significant statistical difference between this group with Lung cancer group (p＜0.05).
