# Supplementary material for: The combination of a seven-autoantibody panel with computed tomography scanning can enhance the diagnostic efficiency of non-small cell lung cancer
Source: Front Oncol. 2022 Nov 30;12:1047019. doi: 10.3389/fonc.2022.1047019 (PMC9748614; doi:10.3389/fonc.2022.1047019)
Supplement: Supplementary file 3 [file Table_3.docx]

**Table S3. Diagnostic performance of various diagnostic methods in different subgroups in the validation set**

| Validation set | | | | | | | | | | | | | |
| --- | --- | --- | --- | --- | --- | --- | --- | --- | --- | --- | --- | --- | --- |
|  |  | Lung cancer | Benign |  |  |  |  |  |  |  |  |  |  |
| 7-AABs  （N=973） | Positive | 317 | 25 | Sen | 0.42 |  |  |  |  |  |  |  |  |
|  | negative | 438 | 193 | spe | 0.89 |  |  |  |  |  |  |  |  |
|  |  |  |  | PPV | 0.91 |  |  |  |  |  |  |  |  |
| CT（N=973） | Malignant | 496 | 52 | Sen | 0.66 |  |  |  |  |  |  |  |  |
|  | Benign | 38 | 61 | spe | 0.28 |  |  |  |  |  |  |  |  |
|  | Unsure | 221 | 105 | PPV | 0.57 |  |  |  |  |  |  |  |  |
| PET-CT（N=101） | Malignant | 71 | 14 | Sen | 0.97 |  |  |  |  |  |  |  |  |
|  | Benign | 2 | 14 | spe | 0.50 |  |  |  |  |  |  |  |  |
|  |  |  |  | PPV | 0.84 |  |  |  |  |  |  |  |  |
| CT+7-AABs（N=973） | Malignant | 302 | 10 | Sen | 0.40 |  |  |  |  |  |  |  |  |
|  | Benign | 30 | 44 | spe | 0.95 |  |  |  |  |  |  |  |  |
|  | Unsure | 423 | 164 | PPV | 0.96 |  |  |  |  |  |  |  |  |
|  |  | ≤8mm | | | | 8<GGO≤20mm | | | | >20mm | | | |
|  |  | Lung cancer | Benign |  |  | Lung cancer | Benign |  |  | Lung cancer | Benign |  |  |
| 7-AABs | Positive | 49 | 11 | Sen | 0.27 | 138 | 11 | Sen | 0.38 | 111 | 3 | Sen | 0.53 |
|  | negative | 133 | 87 | spe | 0.89 | 226 | 77 | spe | 0.88 | 98 | 29 | spe | 0.91 |
|  |  |  |  | PPV | 0.82 |  |  | PPV | 0.90 |  |  | PPV | 0.97 |
| CT | Malignant | 47 | 13 | Sen | 0.26 | 258 | 27 | Sen | 0.71 | 191 | 12 | Sen | 0.91 |
|  | Benign | 10 | 26 | spe | 0.27 | 21 | 24 | spe | 0.27 | 7 | 11 | spe | 0.34 |
|  | Unsure | 125 | 59 | PPV | 0.19 | 85 | 37 | PPV | 0.63 | 11 | 9 | PPV | 0.86 |
| PET-CT | Malignant | 1 | 4 | Sen | 1.00 | 21 | 6 | Sen | 0.95 | 49 | 4 | Sen | 0.98 |
|  | Benign | 0 | 4 | spe | 0.50 | 1 | 6 | spe | 0.50 | 1 | 4 | spe | 0.50 |
|  |  |  |  | PPV | 0.20 |  |  | PPV | 0.78 |  |  | PPV | 0.92 |
| CT+7-AABs | Malignant | 48 | 3 | Sen | 0.26 | 131 | 6 | Sen | 0.36 | 106 | 1 | Sen | 0.51 |
|  | Benign | 9 | 17 | spe | 0.97 | 16 | 18 | spe | 0.93 | 5 | 9 | spe | 0.97 |
|  | Unsure | 125 | 78 | PPV | 0.94 | 217 | 64 | PPV | 0.94 | 98 | 22 | PPV | 0.99 |
|  |  | Pure GGO | | | | Mix GGO | | | | Solid nodule | | | |
|  |  | Lung cancer | Benign |  |  | Lung cancer | Benign |  |  | Lung cancer | Benign |  |  |
| 7-AABs | Positive | 9 | 2 | Sen | 0.18 | 159 | 10 | Sen | 0.38 | 144 | 13 | Sen | 0.50 |
|  | negative | 40 | 25 | spe | 0.93 | 260 | 76 | spe | 0.88 | 143 | 91 | spe | 0.88 |
|  |  |  |  | PPV | 0.82 |  |  | PPV | 0.92 |  |  | PPV | 0.90 |
| CT | Malignant | 22 | 5 | Sen | 0.45 | 250 | 24 | Sen | 0.60 | 224 | 23 | Sen | 0.78 |
|  | Benign | 5 | 6 | spe | 0.22 | 14 | 15 | spe | 0.17 | 19 | 40 | spe | 0.38 |
|  | Unsure | 22 | 16 | PPV | 0.34 | 155 | 47 | PPV | 0.53 | 44 | 41 | PPV | 0.67 |
| PET-CT | Malignant | 0 | 0 | Sen | - | 10 | 2 | Sen | 0.91 | 60 | 12 | Sen | 0.98 |
|  | Benign | 0 | 0 | spe | - | 1 | 1 | spe | 0.33 | 1 | 13 | spe | 0.52 |
|  |  |  |  | PPV | - |  |  | PPV | 0.83 |  |  | PPV | 0.83 |
| CT+7-AABs | Malignant | 7 | 1 | Sen | 0.14 | 155 | 5 | Sen | 0.37 | 138 | 4 | Sen | 0.48 |
|  | Benign | 3 | 4 | spe | 0.96 | 13 | 10 | spe | 0.94 | 14 | 30 | spe | 0.96 |
|  | Unsure | 39 | 22 | PPV | 0.88 | 251 | 71 | PPV | 0.96 | 135 | 70 | PPV | 0.96 |
|  |  | BL | | | | BT | | | | AAH | | | |
|  |  | Lung cancer | Benign |  |  | Lung cancer | Benign |  |  | Lung cancer | Benign |  |  |
| 7-AABs | Positive | 0 | 16 | Sen | - | 0 | 5 | Sen | - | 0 | 4 | Sen |  |
|  | negative | 0 | 105 | spe | 0.87 | 0 | 19 | spe | 0.79 | 0 | 66 | spe | 0.94 |
|  |  |  |  | PPV | - |  |  | PPV | - |  |  | PPV | - |
| CT | Malignant | 0 | 28 | Sen | - | 0 | 3 | Sen | - | 0 | 21 | Sen | - |
|  | Benign | 0 | 46 | spe | 0.38 | 0 | 5 | spe | 0.21 | 0 | 8 | spe | 0.11 |
|  | Unsure | 0 | 47 | PPV | - | 0 | 16 | PPV | - | 0 | 41 | PPV | - |
| PET-CT | Malignant | 0 | 11 | Sen | - | 0 | 2 | Sen | - | 0 | 1 | Sen | - |
|  | Benign | 0 | 10 | spe | 0.48 | 0 | 1 | spe | 0.33 | 0 | 3 | spe | 0.75 |
|  |  |  |  | PPV | - |  |  | PPV | - |  |  | PPV | - |
| CT+7-AABs | Malignant | 0 | 6 | Sen | - | 0 | 1 | Sen | - | 0 | 3 | Sen | - |
|  | Benign | 0 | 34 | spe | 0.95 | 0 | 1 | spe | 0.96 | 0 | 7 | spe | 0.96 |
|  | Unsure | 0 | 81 | PPV | - | 0 | 22 | PPV | - | 0 | 60 | PPV | - |
|  |  | MIA or AIS | | | | IAC | | | | SCC | | | |
|  |  | Lung cancer | Benign |  |  | Lung cancer | Benign |  |  | Lung cancer | Benign |  |  |
| 7-AABs | Positive | 61 | 0 | Sen | 0.29 | 191 | 0 | Sen | 0.40 | 32 | 0 | Sen | 0.62 |
|  | negative | 146 | 0 | spe | - | 287 | 0 | spe | - | 20 | 0 | spe | - |
|  |  |  |  | PPV | - |  |  | PPV | - |  |  | PPV | - |
| CT | Malignant | 74 | 0 | Sen | 0.36 | 361 | 0 | Sen | 0.76 | 47 | 0 | Sen | 0.90 |
|  | Benign | 14 | 0 | spe | - | 23 | 0 | spe | - | 1 | 0 | spe | - |
|  | Unsure | 119 | 0 | PPV | - | 94 | 0 | PPV | - | 4 | 0 | PPV | - |
| PET-CT | Malignant | 0 | 0 | Sen | - | 46 | 0 | Sen | 0.96 | 23 | 0 | Sen | 1.00 |
|  | Benign | 0 | 0 | spe | - | 2 | 0 | spe | - | 0 | 0 | spe | - |
|  |  |  |  | PPV | - |  |  | PPV | - |  |  | PPV | - |
| CT+7-AABs | Malignant | 59 | 0 | Sen | 0.29 | 186 | 0 | Sen | 0.39 | 32 | 0 | Sen | 0.62 |
|  | Benign | 12 | 0 | spe | - | 17 | 0 | spe | - | 1 | 0 | spe | - |
|  | Unsure | 136 | 0 | PPV | - | 275 | 0 | PPV | - | 19 | 0 | PPV | - |
|  |  | SCLC | | | |  |  |  |  |  |  |  |  |
|  |  | Lung cancer | Benign |  |  |  |  |  |  |  |  |  |  |
| 7-AABs | Positive | 6 | 0 | Sen | 0.4 |  |  |  |  |  |  |  |  |
|  | negative | 9 | 0 | spe | - |  |  |  |  |  |  |  |  |
|  |  |  |  | PPV | - |  |  |  |  |  |  |  |  |
| CT | Malignant | 12 | 0 | Sen | 0.80 |  |  |  |  |  |  |  |  |
|  | Benign | 0 | 0 | spe | - |  |  |  |  |  |  |  |  |
|  | Unsure | 3 | 0 | PPV | - |  |  |  |  |  |  |  |  |
| PET-CT | Malignant | 12 | 0 | Sen | 1.00 |  |  |  |  |  |  |  |  |
|  | Benign | 0 | 0 | spe | - |  |  |  |  |  |  |  |  |
|  |  |  |  | PPV | - |  |  |  |  |  |  |  |  |
| CT+7-AABs | Malignant | 6 | 0 | Sen | 0.4 |  |  |  |  |  |  |  |  |
|  | Benign | 0 | 0 | spe | - |  |  |  |  |  |  |  |  |
|  | Unsure | 9 | 0 | PPV | - |  |  |  |  |  |  |  |  |
