# Supplementary figures and images for: The combination of a seven-autoantibody panel with computed tomography scanning can enhance the diagnostic efficiency of non-small cell lung cancer
Source: Front Oncol. 2022 Nov 30;12:1047019. doi: 10.3389/fonc.2022.1047019 (PMC9748614; doi:10.3389/fonc.2022.1047019)

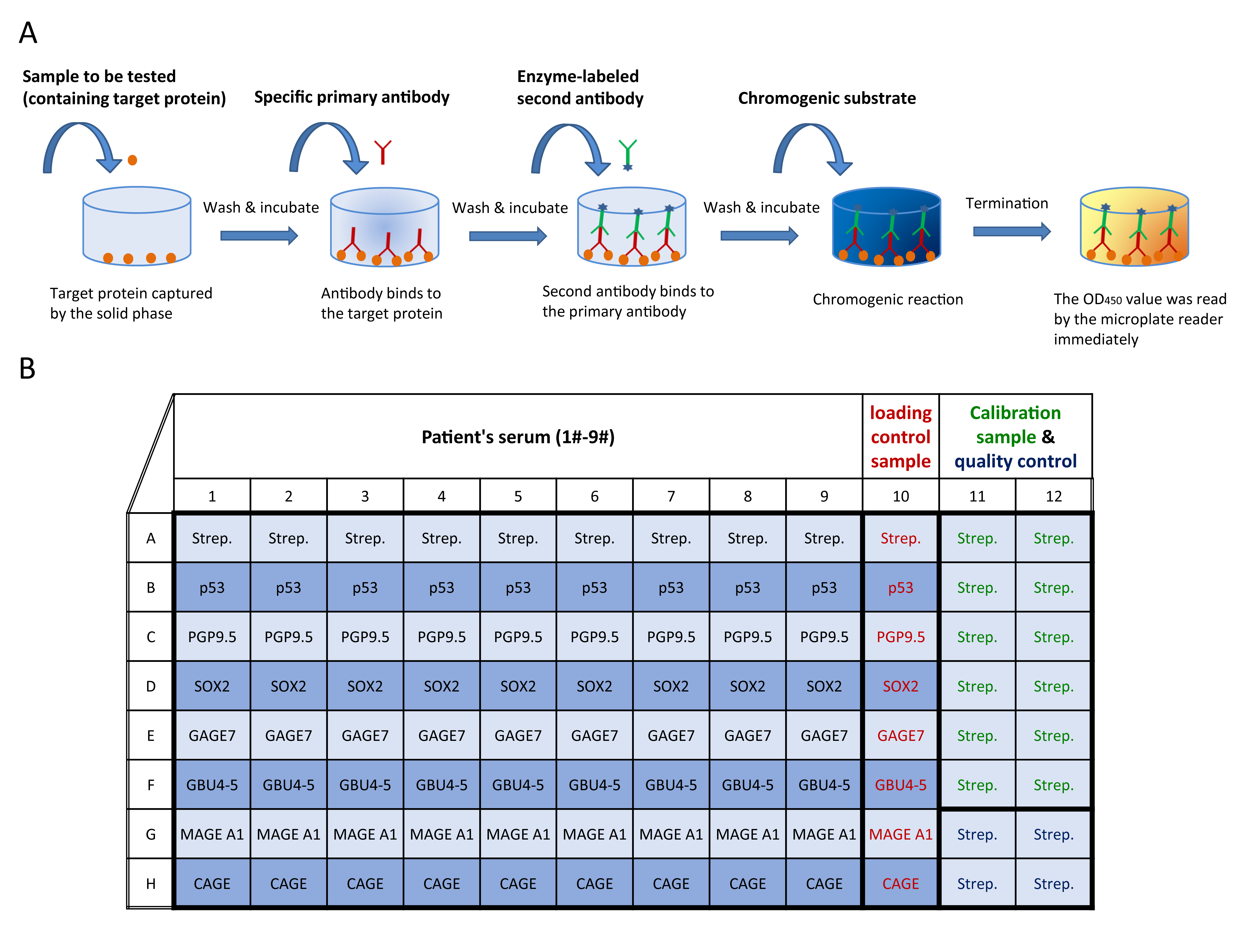

Supplement: Supplementary Figure 1 — Schematic diagram of 7-AABs detection. (A) Procedure for 7-AABs detection by indirect ELISA assay; (B) Setting of patient’s serum samples, loading control samples, calibration samples and quality control samples during 7-AAB panel testing. [file Image_1.tif]
